# The importance of realistic dispersal models in conservation planning: application of a novel modelling platform to evaluate management scenarios in an Afrotropical biodiversity hotspot

**DOI:** 10.1111/1365-2664.12643

**Published:** 2016-03-31

**Authors:** Job Aben, Greta Bocedi, Stephen C. F. Palmer, Petri Pellikka, Diederik Strubbe, Caspar Hallmann, Justin M. J. Travis, Luc Lens, Erik Matthysen

**Affiliations:** ^1^ Institute of Biological and Environmental Sciences University of Aberdeen Zoology Building Tillydrone Avenue Aberdeen AB24 2TZ UK; ^2^ Evolutionary Ecology Group Department of Biology University of Antwerp Groenenborgerlaan 171 2020 Antwerp Belgium; ^3^ Department of Geosciences and Geography University of Helsinki P.O. Box 64 00014 Helsinki Finland; ^4^ Institute of Water and Wetland Research Department of Experimental Plant Ecology Radboud University P. O. Box 9010 6500 GL Nijmegen The Netherlands; ^5^ Terrestrial Ecology Unit Department of Biology Ghent University K.L. Ledeganckstraat 35 9000 Ghent Belgium

**Keywords:** connectivity, conservation planning, demography, dispersal, Eastern Arc Mountains, fragmentation, habitat network, *Phyllastrephus cabanisi*, RangeShifter, SEPM

## Abstract

1. As biodiversity hotspots are often characterized by high human population densities, implementation of conservation management practices that focus only on the protection and enlargement of pristine habitats is potentially unrealistic. An alternative approach to curb species extinction risk involves improving connectivity among existing habitat patches. However, evaluation of spatially explicit management strategies is challenging, as predictive models must account for the process of dispersal, which is difficult in terms of both empirical data collection and modelling.
2. Here, we use a novel, individual‐based modelling platform that couples demographic and mechanistic dispersal models to evaluate the effectiveness of realistic management scenarios tailored to conserve forest birds in a highly fragmented biodiversity hotspot. Scenario performance is evaluated based on the spatial population dynamics of a well‐studied forest bird species.
3. The largest population increase was predicted to occur under scenarios increasing habitat area. However, the effectiveness was sensitive to spatial planning. Compared to adding one large patch to the habitat network, adding several small patches yielded mixed benefits: although overall population sizes increased, specific newly created patches acted as dispersal sinks, which compromised population persistence in some existing patches. Increasing matrix connectivity by the creation of stepping stones is likely to result in enhanced dispersal success and occupancy of smaller patches.
4. *Synthesis and applications*. We show that the effectiveness of spatial management is strongly driven by patterns of individual dispersal across landscapes. For species conservation planning, we advocate the use of models that incorporate adequate realism in demography and, particularly, in dispersal behaviours.

## Abstract 2

We show that the effectiveness of spatial management is strongly driven by patterns of individual dispersal across landscapes. For species conservation planning, we advocate the use of models that incorporate adequate realism in demography and, particularly, in dispersal behaviours.

## Introduction

Habitat loss and fragmentation are key drivers of global biodiversity loss, and their impact is highest in biodiversity hotspots as more species are affected (Brooks *et al*. 2002). Concurrently, these areas are also characterized by high human densities (Cincotta, Wisnewski & Engelman 2000), challenging the implementation of effective conservation management strategies. In particular, the application of traditional top‐down conservation approaches focusing on the protection (and enlargement) of large habitat areas is severely constrained by land availability and socio‐economic issues (Balmford *et al*. 2001). A more promising option for maintaining species in these areas is effectively linking multiple subpopulations by facilitating dispersal, allowing them to function as one larger, more resilient population (i.e. the creation of ‘habitat networks’; Boscolo & Metzger 2011; Baguette *et al*. 2013). As network functioning primarily depends on dispersal rates of individuals among habitat patches, management strategies should aim at reducing patch isolation, either by increasing aggregation (i.e. reducing inter‐patch distances by increasing habitat extent at the landscape level; Hodgson *et al*. 2009) or by improving connectivity of the matrix by creation of corridors (Levey *et al*. 2005) or stepping stones (Fischer & Lindenmayer 2002; Leidner & Haddad 2011).

To assess and prioritize alternative management strategies, spatially explicit population models (SEPMs) have proven to be of high value (e.g. Conlisk *et al*. 2014; Fordham *et al*. 2014). Such models may inform conservation planners by integrating habitat and demographic modelling with the objective of projecting population trends for alternative scenarios (Pe'er *et al*. 2013). From these projections, an estimate may be derived of the (relative) probability of a population persisting to a given future time (Pe'er *et al*. 2013). However, when modelling population dynamics in heterogeneous landscapes, it is important to acknowledge that individuals may occur in patches of different habitat quality, size and geographical position. Moreover, SEPMs need to account for the process of dispersal, which represents a challenge both in terms of acquiring informative empirical data and of requiring more realistic representation of the process in models (Travis *et al*. 2012). The use of simple distance‐dependent functions to estimate dispersal probabilities in most spatially explicit population viability models (Vos *et al*. 2001; Moilanen & Nieminen 2002) may reflect these difficulties. In so doing, these models neglect possible effects of context‐dependent emigration and settlement and of landscape properties, such as matrix composition and configuration, patch size and configuration, on functional landscape connectivity, despite many studies having demonstrated their likely effects on functional connectivity (Ferreras 2001; Bender & Fahrig 2005; Bocedi *et al*. 2014b) and population dynamics (Revilla & Wiegand 2008). Hence, when the objective is to increase species persistence through the creation of habitat networks, moving beyond simple distance measures and incorporating an ecologically more realistic understanding of the dispersal process, and hence of connectivity, into predictive models appears indispensable (Russell, Swihart & Feng 2003; Doerr, Barrett & Doerr 2011; Vasudev & Fletcher 2015).

RangeShifter is a recently developed individual‐based SEPM (Bocedi *et al*. 2014a) which accounts explicitly for the three phases of dispersal (emigration, transfer, settlement). A major benefit is that the distribution of dispersal distances becomes an emergent property of behavioural rules at each phase in interaction with the landscape. For actively dispersing organisms, realized dispersal distances can differ substantially depending on landscape characteristics, yielding potentially major implications for demography (Mennechez, Schtickzelle & Baguette 2003; Bocedi *et al*. 2014b). We here apply RangeShifter to evaluate the effectiveness of a set of realistic spatially explicit management scenarios. Scenarios were developed for a highly anthropogenic landscape in the Eastern Arc Mountains (EAM) biodiversity hotspot (Brooks *et al*. 2002) with the aim to improve persistence of bird species living in cloud forest remnants. The likely scenario performance is evaluated based on the simulated spatial population dynamics of the Cabanis's greenbul *Phyllastrephus cabanisi* which ranks among the best studied forest bird species in sub‐Saharan Africa. Drawing on the experience of developing the model and its predictions, we are able to provide some clear and general recommendations for how dispersal should best be modelled in any actively dispersing organism for which spatial management recommendations are sought.

## Materials and methods

### Study Area

**Figure 1:**
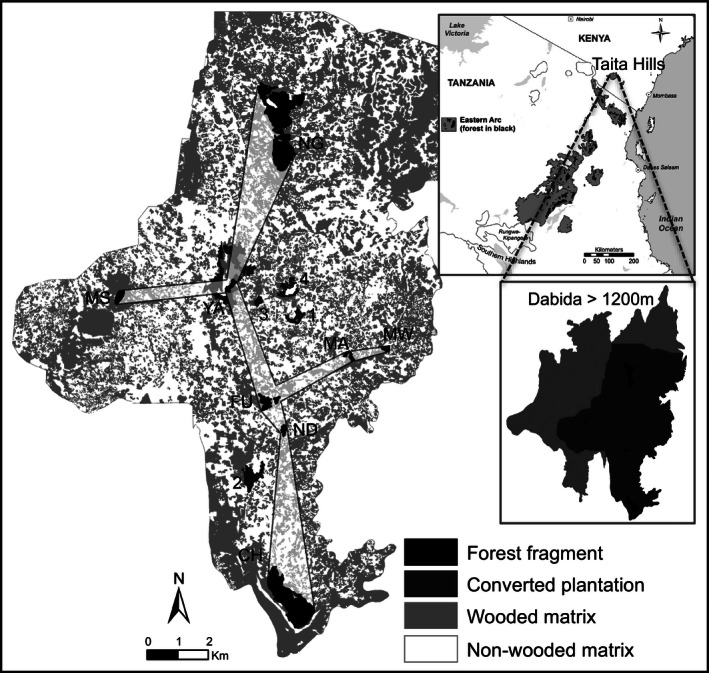
Location of forest fragments and exotic plantations which were converted to habitat patches under scenarios EXOTIC‐S (1 [MB], 2 [SU], 3 [WM], 4 [WR]) and EXOTIC‐L (5 [YA]). For clarity, we show relatively permeable matrix land‐cover types (indigenous forest, exotic plantation, agroforestry and bush) in grey and less permeable types in white (agricultural fields) (see Fig. S1 for a colour map). The transparent zones connecting patches indicate corridors used for the creation of stepping stones under scenario MATRIX‐C. The total area considered for the simulations was 145·6 km^2^ (dark grey in lower inset). Map of EAM modified from Platts *et al*. (2011).

We conducted our analysis in the Dabida massif of the Taita Hills (03°20′S, 38°15′E, alt 1200–2208 m, Fig. 1), the northern‐most mountain block of the EAM. This massif supports several greenbul populations in discrete indigenous forest fragments that are effectively isolated from populations on other massifs by stretches of dry lowland habitat (Callens *et al*. 2011). Like other EAM massifs (Green *et al*. 2013), the indigenous forest extent on Dabida has been drastically reduced by intense human pressures on land. Nowadays, forest fragments large enough to host viable greenbul populations cover only 256 ha (Pellikka *et al*. 2009) (median 5·2 ha, Table 1). Vegetation surveys showed that only the largest fragment (NG) represented high‐quality forest habitat, all others being degraded (Aerts *et al*. 2011). Given observed area requirements of the species (below), only patches >2 ha (*n* = 8) were treated as suitable breeding habitat (Fig. 1, Table 1). Forest fragments are embedded in a matrix characterized by the presence of smallholder cultivation plots, exotic plantations and human settlements (Pellikka *et al*. 2009).

**Table 1:** Summary of model parameters

Model parameter	Symbol	Estimate
Density dependence coefficient 1/b (individuals per ha)		
NG (126 ha)		28·986
CH (89·5 ha)		23·256
FU (10 ha)		23·256
MA (2·7 ha)		23·256
MS (6·4 ha)		23·256
MW (2·3 ha)		23·256
ND (3 ha)		23·256
YA (4 ha)		23·256
Proportion of males at birth		0·50
Mean fecundity	φ	1·38
Survival rates		
Male juvenile	σ_0m_	0·508
Female juvenile	σ_0f_	0·463
Male adult	σ_1m_	0·850
Female adult	σ_1f_	0·825
Emigration probability	*d*	
Max. emigration probability male juvenile	*D*_0m_	0·50
Max. emigration probability female juvenile	*D*_0f_	0·70
α male juvenile		0·60
β male juvenile		0·094
α female juvenile		0·60
β female juvenile		0·093
Movement parameters		
Directional persistence	DP	2
Perceptual range	PR	20 grid cells
Memory size	MS	2
Dispersal bias	DB	1·1
Settlement probability	*P*_s_	
α male juvenile		−150
β male juvenile		0·055
Cost surface		
Indigenous forest		1
Exotic plantation forest		24
Agroforestry		12
Bush		25
Field (agricultural)		77
Built‐up		7700
Initial density (individuals per ha) in all fragments		1·225

### Model Species

Cabanis's greenbul is an insectivorous passerine of montane forest understorey. Comprehensive data are available on survival rates (Korfanta, Newmark & Kauffman 2012), patch occupancy (Newmark 1991; Lens *et al*. 2002), sensitivity to forest disturbance (Lens *et al*. 1999; Newmark 2006), population genetic structure and gene flow (Callens *et al*. 2011; Coulon *et al*. 2015), and land‐cover selection while moving through the matrix (Aben *et al*. 2012). In the study area, the species has been reliably recorded in all indigenous forest fragments, although successful breeding has hitherto been confirmed only in the largest two (CH, NG) and three smaller (FU, MS, ND) fragments.

### Modelling Population Dynamics Using RangeShifter

We modelled stage‐structured population dynamics at the scale of individual patches according to vital rates assigned to juvenile (age <1 year) and adult (age >1 year) stages. After reproduction, juveniles could emigrate depending on local density. After all dispersers settled in new breeding patches, stage‐specific survival probability was applied. Surviving juveniles then became adults. We modelled one reproductive event per year; the number of offspring produced by each female was sampled from a Poisson distribution with mean fecundity of 1·38 fledglings per pair per season (Appendix S1 in Supporting information). The fledgling sex ratio was 0·5, as there was no sex bias in 63 nests with 2 pulli (Lens, unpublished data). Sex‐specific survival rates for juveniles and adults were estimated from long‐term (1996–2010) capture–mark–recapture data using the Cormack–Jolly–Seber method (MARK; White & Burnham 1999) (Appendix S2).

We modelled the density‐dependent survival probability of stage *i* and sex *s*, σ_is_, as:

(eqn 1) $$\sigma_{\mathrm{is}}\;=\;\sigma_{0,\mathrm{is}}*e^{-bN_{t}}$$

where *σ*_0,is_ is the survival probability at low densities, *b* is the strength of density dependence, and *N*_*t*_ is the local population density at time *t*. The RangeShifter parameter 1/*b* was estimated such that emergent equilibrium population densities reflected currently observed densities, namely 1·36 and 1·09 individuals ha^−1^ for the high‐quality patch and the degraded patches, respectively (Appendix S1), by fitting equation 1 to assumed survival rates at low density and the estimated survival rates at current density for both high‐ and low‐quality patches. We assumed survival rates at low density to be 5% above average rates, that is weak density‐dependent survival. As estimated population density differed according to patch quality, distinct values for 1/*b* were assigned to patches (Table 1), which provided the expected equilibrium density for both patch types.

Emigration was modelled as the stage‐, sex‐ and density‐dependent probability (*d*) that an individual leaves its natal patch (Appendix S3). Only juveniles could emigrate, at *d *=* *0·20 for females and *d *=* *0·15 for males at baseline population densities. While we have no empirical estimates, these values were chosen because they resulted on average in nine dispersers per year under current landscape conditions. This seems realistic given that our intensive capture–recapture studies detected 1·2 dispersers per year (only birds ringed as nestling or immature), which undoubtedly is an underestimate.

Movement of dispersers was modelled using the stochastic movement simulator (SMS; Palmer, Coulon & Travis 2011) embedded in RangeShifter. SMS simulates discrete individual stepwise nearest‐neighbour movements across a cost surface until individuals settle in a non‐natal habitat patch or die after a specified maximum number of steps or because of per‐step mortality risk. Here, we used a 5‐m‐resolution surface where cost values inversely reflect the relative preference of Cabanis's greenbuls for the matrix land‐cover types (Aben *et al*. 2012; Coulon *et al*. 2015; Table 1; Fig S1). The movement probability to each neighbouring cell is determined by the cost value of that cell (and the surrounding cells within the individual's perceptual range) and by the individual's tendency to follow a straight line (i.e. directional persistence, DP). Here, we used a modified version having two additional SMS parameters to control the number of steps used for calculating DP (i.e. memory size) and the degree of effective displacement of dispersers relative to their natal patch (i.e. dispersal bias; Coulon *et al*. 2015). We previously demonstrated that SMS accurately predicted actual movement paths (Aben *et al*. 2014) and genetic estimates of inter‐patch connectivity of Cabanis's greenbuls (Coulon *et al*. 2015), from which studies parameter estimates for DP, memory size and dispersal bias were obtained (Table 1). We assumed a perceptual range of 100 m, which corresponds to the maximum observed distance individuals were willing to cross the non‐forested matrix in a single flight (Aben *et al*. 2012). Dispersers were allowed 10^6^ steps and assigned a quasi‐zero per‐step mortality probability such that dispersers could move even between the two most distant patches.

Upon encountering a non‐natal patch, males settled with a probability inversely related to the local density (eqn. S3·2), while female settlement was assumed to be constrained by the availability of at least one potential mate (Appendix S3).

### Management Scenarios

Population dynamics were simulated for the current (baseline) landscape and for three realistic conservation management scenarios earlier formulated during workshops with multiple stakeholders (e.g. policymakers, scientists, the local community) (Githiru *et al*. 2011): (i) *forest restoration*: augmenting existing forest patches with indigenous trees; (ii) *matrix enrichment*: planting indigenous tree species at homesteads; and (iii) *exotics conversion*: conversion of exotic plantations to indigenous forest (Table 2).

**Table 2:** Summary of the simulated management scenarios. Expected population size is a function of total area of suitable habitat and estimates of current population densities

Scenario	Number of patches	Total area of breeding habitat (ha)	Expected population size	Mean (range) of inter‐patch distance to nearest patch (m)
Baseline	8	244	300	1948 (622–3676)
RESTORE	8	244	332	1948 (622–3676)
MATRIX‐R	8	244	300	1948 (622–3676)
MATRIX‐C	8	244	300	1948 (622–3676)
EXOTIC‐S	12	296	357	1135 (350–3027)
EXOTIC‐L	8	299	360	1701 (622–3676)

In a first scenario (RESTORE), we simulated the effect of restoring all currently degraded indigenous forest patches to their natural state. This was achieved by assuming a density dependence coefficient 1/*b* for these patches equal to that of the highest quality patch NG (Table 2).

In a second set of scenarios, we simulated planting indigenous vegetation around houses (total area approximately 55 ha). This was achieved by changing the matrix cost values within a 5 m radius around selected houses to the value of indigenous forest, thereby enhancing connectivity when these low‐cost areas are used as stepping stones by dispersers. This was justified by detailed observations of translocated birds indicating that the direct vicinity of houses was not avoided, but was simply unattractive when lacking trees (Aben *et al*. 2012). In MATRIX‐R, we randomly selected 25% of all homesteads for indigenous tree planting within a minimum convex polygon encapsulating all habitat patches. In MATRIX‐C, we selected homesteads only within corridors connecting each habitat patch to the habitat network at a minimum cumulative length (Fig. 1, Table 2).

In a third set of scenarios, we simulated conversion of five exotic plantations (currently lacking greenbul populations) to indigenous forests (total area approximately 55 ha) based on selection criteria formulated in Githiru *et al*. (2011). In EXOTIC‐S, we modelled conversion of four small plantations (i.e. MB [10 ha], SU [22 ha], WM [4·3 ha], WR [16 ha]) while in EXOTIC‐L, we simulated conversion of a single large plantation (YA [55 ha]) (Fig. 1, Table 2). In the latter case, the selected plantation bordered an existing habitat patch; here, the two were merged yielding a single habitat patch of 59 ha (YA). Converted exotic plantations were assigned a coefficient 1/*b* equal to that of current degraded forest habitat, as it is uncertain that converted patches would achieve optimal habitat quality.

### Initialization and Evaluation

All simulations were initialized according to current observed patch occupancy patterns. Patches MA, MW and YA and all newly created habitat patches in the exotics conversion scenarios were initially vacant. All other patches were initialized at 1·225 individuals ha^−1^ (the mean of the estimated current densities).

For each scenario, we simulated population dynamics for 50 years and ran 100 replicates of each. We calculated three measures relevant for evaluating effects of variation in landscape configuration and hence in the functioning of the network: average dispersal success (proportion of emigrants successfully settling in a non‐natal habitat patch), average patch‐level number of immigrants per year and patch‐level occupancy probability (the proportion of replicates in which the patch was occupied) averaged over all years. Additionally, the final year population, averaged over the replicates, was used to evaluate the overall performance of the five scenarios.

Finally, for each scenario, we performed a sensitivity analysis to assess the impact of four key input parameters (adult survival, juvenile survival, fecundity, each varied by ± 10%; maximum emigration probability, ± 20%) on the expected final population size (Appendix S4).

## Results

### Total Population Size

**Figure 2:**
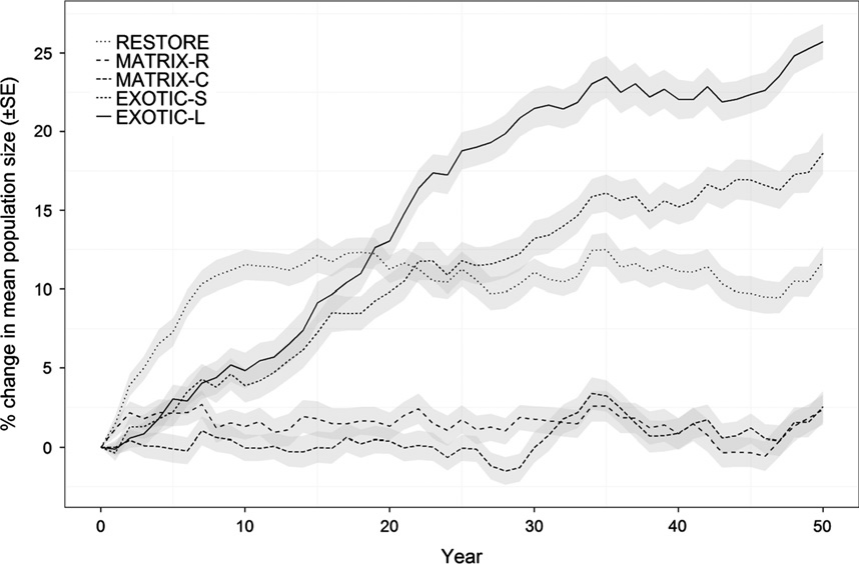
Percentage change in mean projected population size relative to the baseline under five management scenarios: habitat restoration of degraded forest patches (RESTORE), creating stepping stones in the matrix randomly (MATRIX‐R) or within corridors connecting patches (MATRIX‐C), and converting exotic plantations to forest habitat of either several small patches (EXOTIC‐S) or one large patch (EXOTIC‐L).

Average final abundances obtained under the five management scenarios show that converting the exotic plantation YA into indigenous forest (EXOTIC‐L) is likely the most effective strategy for increasing population size (Fig. 2). Creating the same amount of habitat by converting several smaller plantations (EXOTIC‐S) is less effective but still outperforms forest restoration (Fig. 2). Scenarios targeted at improving matrix connectivity only had little effect on the total population size (Fig. 2).

In terms of the total population, EXOTIC‐L remained the highest ranked scenario when fecundity, juvenile survival and maximum emigration probability were varied, or when adult survival was increased (Fig. S4.1). Only when 10% lower adult survival was applied was EXOTIC‐L outperformed by RESTORE. In this case, no scenario did better than +5% compared to the baseline, for which a strong decrease in the total population was predicted (−51%; Fig. S4.1).

### Functioning of the Habitat Network

**Figure 3:**
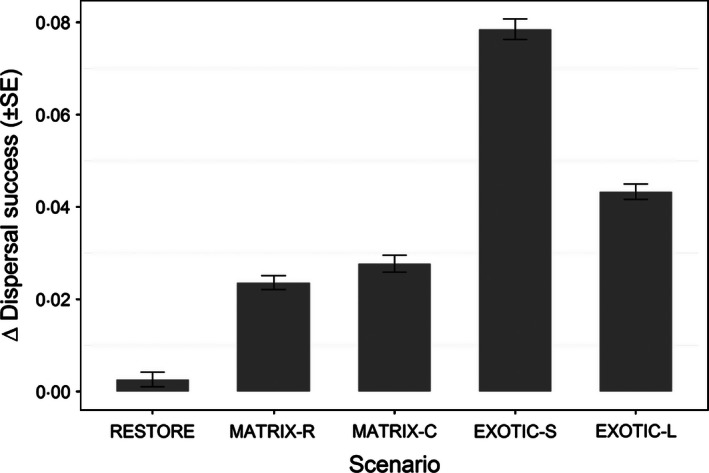
Mean dispersal success (proportion of emigrants successfully settling in a non‐natal patch) in each scenario relative to that of the baseline scenario (i.e. 0·232).

**Figure 4:**
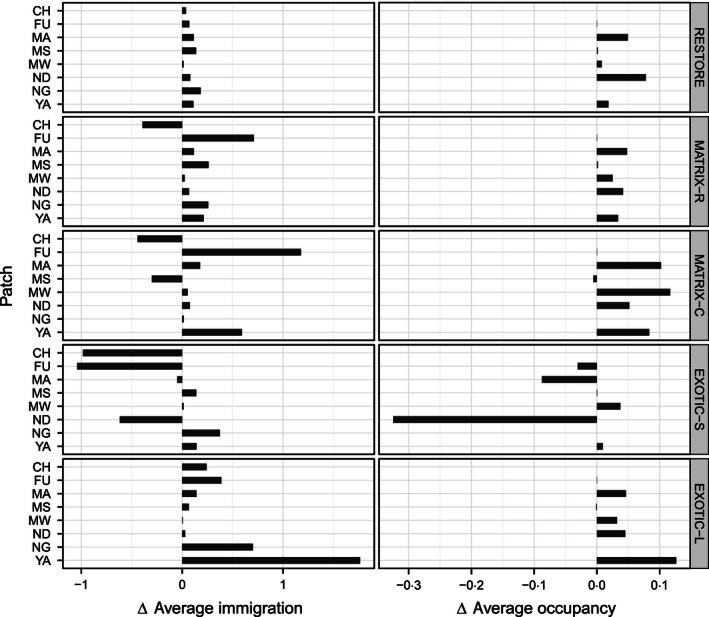
Mean yearly immigration rates and probabilities of patch occupancy for each patch under five management scenarios relative to the baseline scenario (see Table S1). Only patches considered in every simulation are shown.

**Figure 5:**
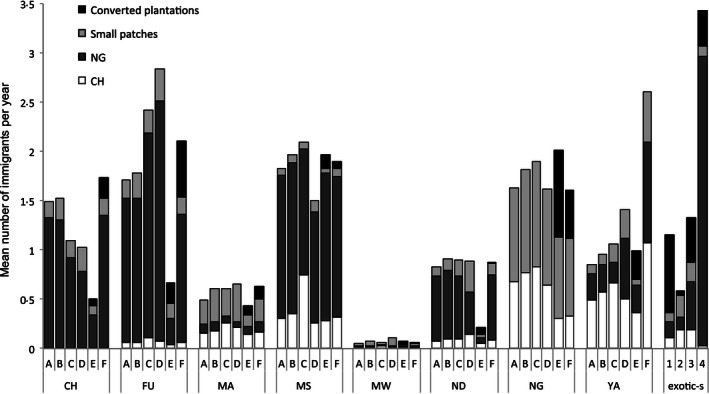
Mean yearly immigration rates for each patch under six scenarios (A [baseline], B [RESTORE], C [MATRIX‐R], D [MATRIX‐C], E [EXOTIC‐S], F [EXOTIC‐L]) and for the four patches converted under the EXOTIC‐S scenario (1 [MB], 2 [SU], 3 [WM], 4 [WR]). Each bar depicts the mean number of immigrants per year originating from a large patch (CH or NG), from one of the smaller existing patches and from a converted plantation.

Overall, dispersal success was predicted to increase under scenarios targeted at improving habitat network functionality (Fig. 3). Patch‐level evaluation showed that, except for some patch–scenario combinations (see below), simulated management also resulted in larger absolute numbers of immigrants per year compared to the baseline (Fig. 4). Planting indigenous trees at homesteads increased immigration rates for all patches except CH (MATRIX‐R) and CH and MS (MATRIX‐C), which were predicted to receive fewer immigrants (Fig. 4). The positive effect of these stepping stone scenarios is largest for the patch located in the centre of the habitat network (FU) and for YA when planting is concentrated in corridors (Fig. 4). The effect of converting plantations on yearly immigration rates varied markedly: an increase was predicted for all patches under EXOTIC‐L while under EXOTIC‐S half of the existing patches were predicted to receive fewer immigrants (Fig. 4). This decrease was particularly large for the southerly patches (Fig. 4) and was caused by a decrease in immigrants originating from NG (Fig. 5). Under this scenario, a large proportion (53%) of dispersers from NG are captured by the newly created patch WR which in turn becomes an important source of immigrants for the nearby smaller patches MB and WM (providing on average 50% of immigrants; Fig. 5). These new patches, however, cannot compensate for the loss in immigrants originating from NG experienced by the other patches in the network: only 33% of that loss is compensated for across all other patches and only 13% for the patches CH, FU and ND (Fig. 5).

Mean patch occupancy was predicted to increase under all scenarios relative to the baseline (Fig. 4) except under EXOTIC‐S (lower for FU, MA and, particularly, ND) and under MATRIX‐C (slightly lower for MS) (Fig. 4). Overall, MATRIX‐C was predicted to have the largest occupancy increases, especially for the two smallest patches (MA and MW) and for YA (Fig. 4).

### Populations in Converted Plantations

**Figure 6:**
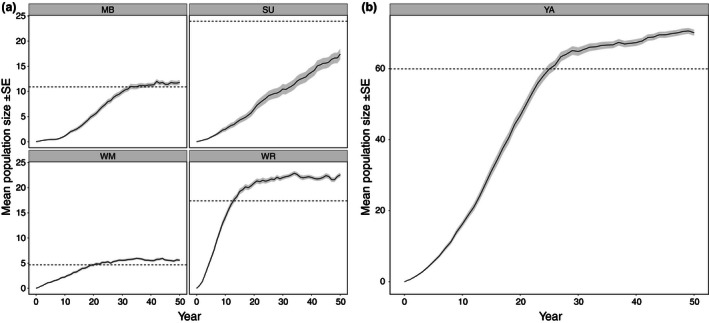
Mean yearly population sizes predicted for the newly created habitat patches under the EXOTIC‐S (a) and EXOTIC‐L (b) scenarios. The dashed line represents the population size expected for each patch assuming that converted plantations can support populations at a density of 1·09 individuals per ha (i.e. the estimated current density in low‐quality forest patches).

All plantations converted under EXOTIC‐S were colonized, but yearly immigration rates differed markedly (Fig. 5). Establishing populations in the converted plantations near the centre of the habitat network (MB, WM, WR) had relatively high growth rates and, after 50 years, reached higher densities than expected (Fig. 6), while SU clearly experienced a low average growth rate (Fig. 6). Patches MB, WM and WR also differed from SU by having lower variation in yearly population sizes across replicates (Fig. 6).

Enlargement of YA under EXOTIC‐L resulted in higher yearly immigration rates and patch occupancy (Figs 4 and 5), as expected. Population growth rate was high especially in the first 30 years and had not completely levelled off by the end of the simulations (Fig. 6).

## Discussion

In this paper, we used recently developed modelling tools to evaluate the effectiveness of a set of alternative management scenarios for spatially structured populations. The novelty of our approach is to include much greater detail in the dispersal process than is typical in PVAs. This allowed us to demonstrate that the effectiveness of conservation management of fragmented landscapes can strongly depend on the spatial configuration of habitat and on the arrangement and density of landscape features that have been added to increase connectivity. Our results therefore illustrate the applicability of integrated spatial models to inform conservation managers about the functioning of ecological networks under both current conditions and for alternative management scenarios.

We evaluated scenarios that differed markedly in the way they were hypothesized to affect greenbul local persistence (i.e. increasing carrying capacity or connectivity). Scenario evaluation based on the total population reflects these fundamental differences; scenarios having a higher carrying capacity (‘habitat scenarios’) are predicted to be more effective compared to the ‘matrix scenarios’. This distinction, however, is accentuated by our study landscape where two large patches harbour 90% of the total population and were not reliant on immigration. Alternative metrics better suited to evaluate functioning of habitat networks, however, provided a more nuanced picture. For instance, occupancy of smaller patches increased as much, or more so, under the matrix scenarios compared to the habitat scenarios. However, outcomes of model simulations were not predictable on the basis of available habitat alone. Final population sizes differed between scenarios having similar available habitat, suggesting an interaction effect between habitat spatial configuration and population dynamics.

For species inhabiting fragmented landscapes, overall population size at a particular time is determined by the combination of patch occupancy and mean population sizes. These patterns are strongly driven by three key parameters: patch area, habitat quality and patch isolation (Thomas *et al*. 2001). In theory, large patches support more persistent populations, populations inhabiting optimal habitat have higher equilibrium densities than those inhabiting suboptimal habitat, and less isolated patches are more likely to be occupied. To these we can now add a fourth effect, namely that of functional connectivity, which is itself influenced by structural connectivity and habitat aggregation.

### Dispersal Success

Variation in expected immigration rates was driven by scenario‐specific degree of structural connectivity, total population size, degree of habitat aggregation, or a combination of the latter two. Since density‐dependent emigration rates did not differ between scenarios, enlarging the population will necessarily increase the number of emigrants and thus the potential for functional connectivity among network patches. This effect was demonstrated by our simulation of habitat restoration in degraded patches, which resulted in a higher number of emigrants relative to the baseline scenario (+7%). The proportion of emigrants actually arriving in a non‐natal patch, however, rather depends on landscape properties and may be regarded a more important factor (versus net production of emigrants) in the persistence of a spatially structured population, especially when dispersal imposes a risk to individuals (Brooker & Brooker 2002; Eklöf, Kaneryd & Munger 2012). Management targeted at optimizing dispersal success may therefore represent an efficient strategy to increase the persistence of spatially structured populations in highly fragmented landscapes. This may be achieved either by reducing inter‐patch distances (here simulated by creating additional habitat patches) or by reducing the costs associated with moving through the matrix (here simulated by creation of stepping stones). Our results are indicative of the effectiveness of both strategies.

Creation of stepping stone corridors resulted on average in higher dispersal success than randomly placed stepping stones. Patch‐level immigration rates revealed that the structural linkages between patches were effective in funnelling the flow of dispersers. For instance, connecting patches NG and YA resulted in strongly increased immigration rates not only in YA but also in FU, which in turn was connected with YA. Thus, positive effects of creating structural linkages may cascade through a habitat network, especially, as here, if dispersers from a large source patch can be funnelled effectively into the network such that a larger proportion survive the transfer phase and contribute to population persistence in smaller patches.

Compared to the effect of stepping stones, increasing habitat aggregation by adding multiple small habitat patches to the network had a much stronger positive effect on overall dispersal success. This scenario in particular differs from all others by reducing inter‐patch distances, thereby increasing the chances for a disperser to locate a new patch (Gustafson & Gardner 1996). However, it was not beneficial for all patches in the network. Conversion of small exotic plantations reduced patch isolation especially in the centre of the network, which in turn resulted in an increase of local dispersal events among these small newly created patches at the cost of a reduction in dispersers to more distant patches. This was caused by a disruption of the flow of dispersers originating from NG on which these patches heavily relied and for which the newly created patches did not compensate. This ‘shading effect’ (*sensu* Hein *et al*. 2004) was likely caused by the two smallest additional patches acting as ‘disperser sinks’. Due to their small size, their populations are likely to have a higher extinction risk, and they must receive immigrants repeatedly before they achieve a stable population and in turn produce dispersers. For FU and in particular ND, shading resulted in strongly elevated subpopulation extinction probabilities.

### Planning of Additional Habitat: The Importance of Immigration

Effectiveness of habitat augmentation depended strongly on the degree to which new habitat was functionally connected. This explained variation in projected population size between the two exotics conversion scenarios; under EXOTIC‐S, a relatively large proportion of additional habitat (21%, patch SU) received too few immigrants to sustain a large persistent population or to counteract negative effects of demographic stochasticity and emigration (reflected by relatively low patch occupancy probabilities), while for EXOTIC‐L, additional habitat was subject to relatively high immigration rates resulting in rapid population growth and a large persistent population. These findings corroborate a recent modelling study which found patches receiving roughly 10% of their carrying capacity annually by immigration performed best (Conlisk *et al*. 2014).

Our results provide a strong and realistic example of the effects of habitat augmentation on spatial population dynamics and clearly show that both the size and location of newly created habitat patches strongly determine their conservation value. For this option to be effective, additional patches must be functionally connected to existing patches and be large enough to host a population capable of producing sufficient dispersers to avoid the risk that they will act as sinks. In line with previous recommendations (Kramer‐Schadt *et al*. 2011; Conlisk *et al*. 2014; Saura, Bodin & Fortin 2014), our results argue for a thorough consideration of the trade‐offs related to size and location when creating additional habitat patches as a conservation measure.

### Synthesis

Creating connected habitat networks is frequently advocated as a viable option for conserving species in fragmented landscapes (e.g. Baguette *et al*. 2013; Villard & Metzger 2014). As effectiveness of such networks greatly depends on how landscape elements promote overall functional connectivity, we demonstrate that alternative network scenarios should be evaluated using SEPMs that can capture realistically the processes that are key drivers of spatial population dynamics. RangeShifter is distinct from commonly used SEPMs in the way dispersal is modelled, and our case study highlights the advantage of this feature in the field of spatial conservation planning.

Of particular importance in this respect is that a mechanistic dispersal model allows evaluation of the effects of changes in landscape structural connectivity on population‐level processes. For instance, by providing output on the number of emigrants and immigrants, RangeShifter allowed us to demonstrate that stepping stones of indigenous vegetation in the matrix are likely to increase dispersal success. These metrics, although potentially of high value to conservation planning, cannot be obtained using conventional SEPMs.

Even more important, inter‐patch dispersal rates are the outcome of context‐dependent dispersal decisions and realistic movement simulations rather than being determined *a priori* by inter‐patch distances. Rates are hence determined by dispersal routes and the size and configuration of habitat patches which together affect the patch encounter probability. Although, in general, our results show that nearby patches are more likely to be connected functionally, our realistic dispersal simulations revealed a strong effect of patch location on degree of functional connectedness, independently from Euclidean distance or size. The ‘shading effect’ of some newly created patches is another interesting result of our study; while theoretical papers have indicated the possibility of such an effect (Hein *et al*. 2004; Pfenning, Hovestadt & Poethke 2004; Heinz *et al*. 2005), our results provide an important demonstration of its potential significance for conservation, further highlighting the value of mechanistic dispersal modelling. In a conventional distance‐based SEPM framework, the addition of new patches results in a deterministic change in between‐patch dispersal probabilities, while our model clearly showed that new patches can have disproportionally large effects on functional connectivity when dispersal probabilities do not follow distance functions. In general, our results strongly underline that the degree by which patches are functionally connected cannot simply be predicted based upon inter‐patch distances. Although this notion is gaining attention in the field of connectivity modelling (Kool, Moilanen & Treml 2013; Coulon *et al*. 2015), availability of SEPMs allowing incorporation of more realistic connectivity measures has hitherto been limited.

Our view is that we should seek to include ‘adequate realism’ in model structure, even where we do not have high‐quality information on parameter estimates. For example, we may have a species for which we have an estimate of its dispersal kernel mean but very little information dispersal behaviour (i.e. emigration, movement or settlement decisions). One option is to build a classical model using the dispersal kernel approach. For some conservation questions, this may turn out to be adequate and recommendations may be robust to the implicit assumptions. However, especially where the question relates to identifying which exact patches of habitat should be conserved or restored, we would strongly advise first also running an alternative model that incorporates the extra mechanistic detail in dispersal in order to explore how robust recommendations are to the structural realism of the model. For those species for which we lack the parameter estimates for dispersal that we possess for the Cabanis's greenbul, then our advice would be to sweep a broad region of plausible parameter space and explore the robustness of the conservation recommendation to parameter uncertainty. A particular strength of RangeShifter is that it facilitates this process of joint model development, as dispersal can be described using kernels as well as using a more mechanistic approach.

Dispersal remains a process that presents challenges in terms of gaining high‐quality data for model parameterization. However, new technologies are resulting in rapidly improving and less costly direct (e.g. GPS) and indirect (e.g. genetics) methods. We are convinced that considerable benefits can be gained by using improved dispersal data to parameterize more realistic models, which can provide more reliable evaluation of spatially explicit management scenarios. By doing so, uncertainty in predictions will be reduced, allowing managers to make better‐informed decisions.

## Data accessibility

All data are presented in online supporting information.

## Supporting information


**Appendix S1.** Estimating fecundity and current population density.
**Appendix S2.** Survival modelling.
**Appendix S3.** Density dependence in dispersal and settlement.
**Appendix S4.** Sensitivity analysis.
**Table S1.** Baseline predictions.
**Fig. S1.** Map of the cost surface.
