# Supplementary material for: The importance of realistic dispersal models in conservation planning: application of a novel modelling platform to evaluate management scenarios in an Afrotropical biodiversity hotspot
Source: J Appl Ecol. 2016 Mar 31;53(4):1055–65. doi: 10.1111/1365-2664.12643 (PMC5042109; doi:10.1111/1365-2664.12643)
Supplement: Supplementary file 1 — Appendix S1. Estimating fecundity and current population density. Appendix S2. Survival modelling. Appendix S3. Density dependence in dispersal and settlement. Appendix S4. Sensitivity analysis. Table S1. Baseline predictions. Fig. S1. Map of the cost surface. [file JPE-53-1055-s001.docx]

*Appendix S1: Estimating fecundity and current population density*

Fecundity was estimated from nest success data (number of fledged individuals/nest) in two fragments (CH 2008-09; NG 2009-10; unpublished data). Nests were located by local research assistants and monitored on a daily basis. In case nests were unsuccessful, multiple nest attempts (up to three) per breeding season were documented. Hence, fecundity was calculated as the average number of fledglings/pair/season (n=45 pairs). Different nests were assigned to a breeding pair according to information on the identity of adult birds associated with the nests, the nest initiation date and between-nest distances.

In order to obtain an estimate of current population density we used detailed data on nest locations and the identity of associated individuals to delineate non-overlapping territories in the fragments CH and NG (recorded in 2008-09 and 2009-10, respectively). An estimate of current population density was obtained by multiplying the number of territories by the average number of birds associated with a territory ([Callens 2012](#_ENREF_3)). This yielded an estimated density of 1.36 and 1.09 individuals/ha for NG and CH respectively. No data were available to estimate territory density for the smaller fragments. However, detailed vegetation surveys carried out in cloud forest fragments on Dabida ([Aerts et al. 2011](#_ENREF_1)) showed that these smaller fragments were more similar to CH (characterized as degraded forest habitat) than to NG (characterized as high quality forest habitat) in terms of habitat quality. Accordingly, these patches were assumed to have a similar territory density as CH and were also assigned a density of 1.09 inds/ha.

*Appendix S2: Survival modelling*

Sex and age specific survival rates for the Cabanis’s greenbul were estimated using 15 years of mark recapture data across five fragments in the Taita Hills based on routine mist-netting sessions. Data was pooled into monthly recapture occasions. Missing sampling occasions were explicitly taken into account in out models by specifying a zero-capture probability. In total 142 sampling occasions were created between 1996 and 2011. We used Cormack Jolly Seber models to estimate age, gender, and fragment specific estimates of survival from the ring recoveries, using program MARK ([White and Burnham 1999](#_ENREF_5)). We differentiated survival between gender (male, female) and age (adult and immatures). Our most general model included gender, fragment and age effects along with all possible interactions for apparent survival, and gender, fragment and interaction effects for the recapture rates. Because of data sparseness, we did not consider time dependency in either survival or recapture rates. We evaluated all possible nested models (n=70) within our general model. We compared relative fit of our nested models using Akaike’s Information Criterion adjusted for sample size and overdispersion ([Burnham and Anderson 2002](#_ENREF_2)). Goodness of fit (GOF) was not assessed on the most global model directly, because missing occasions did not allow using the GOF modules of program MARK. Instead, we examined how the relative ranking of the models changed when assuming higher order of overdispersion (set manually, in increments of 0.25 between 1 and 2.5). As this did not change qualitatively the rankings of the models as judged by the *QAICc* metric, we here report estimates based on an assumed overdispersion factor of *c=*1 (Burnham and Anderson 1991). Because none of the models stood out as most parsimonious (Table S2.1), we used model averaging of our survival parameters in order to incorporate model uncertainty in our final estimates of survival and (Table S2.2). Finally, because RangeShifter not allows varying survival between different fragments, these estimates were averaged over fragments to obtain a single estimate of survival for each age x gender class (Table S2.3).

In total 70 models were run, with models including fragment, age and gender effects for survival and fragment and gender effects for the recapture parameters receiving the highest model support (Table S2.1). Model averaged estimates of survival revealed higher survival for adults over immatures, and slightly higher estimates for males over females (Table S2.2). The survival estimates for Cabanis’s greenbul populations in the Taita Hills closely match those obtained for populations in another EAM mountain bloc, the Usambara Mts. ([Korfanta et al. 2012](#_ENREF_4)).

| **Table S2.1.** Ranking of the 15 (out of 70) most parsimonious CJS models fitted to Cabanisi’s greenbul CMR data in the Taita Hills. For each model, we provide the symbolic description, number of parameters, *AICc* metric, the relative difference in *AICc* units, relative model weight, and deviance. Model ranking is based assuming no overdispersion (i.e. *c*=1*)*. | | | | | |
| --- | --- | --- | --- | --- | --- |
| Model | npar | AICc | DeltaAICc | Weight | Deviance |
| *Phi(~FRAG * age2 + SEX)p(~FRAG + SEX)* | 19 | 11994.14 | 0 | 2.33E-01 | 10218.86 |
| *Phi(~FRAG + age2 + SEX)p(~FRAG + SEX)* | 15 | 11994.33 | 0.1958272 | 2.11E-01 | 10227.16 |
| *Phi(~FRAG + age2 + SEX)p(~FRAG * SEX)* | 23 | 11994.77 | 0.6353327 | 1.70E-01 | 10211.38 |
| *Phi(~FRAG * age2 + SEX)p(~FRAG * SEX)* | 27 | 11994.89 | 0.7589248 | 1.59E-01 | 10203.35 |
| *Phi(~FRAG + age2 * SEX)p(~FRAG + SEX)* | 17 | 11995.82 | 1.6800248 | 1.01E-01 | 10224.6 |
| *Phi(~FRAG + age2 * SEX)p(~FRAG * SEX)* | 25 | 11996.32 | 2.1867151 | 7.80E-02 | 10208.86 |
| *Phi(~age2 + SEX)p(~FRAG * SEX)* | 19 | 11999.58 | 5.444 | 1.53E-02 | 10224.31 |
| *Phi(~age2 + SEX)p(~FRAG + SEX)* | 11 | 11999.86 | 5.7267153 | 1.33E-02 | 10240.77 |
| *Phi(~age2 * SEX)p(~FRAG * SEX)* | 21 | 12001.01 | 6.8737652 | 7.49E-03 | 10221.68 |
| *Phi(~age2 * SEX)p(~FRAG + SEX)* | 13 | 12001.33 | 7.1973948 | 6.37E-03 | 10238.2 |
| *Phi(~FRAG + age2 + SEX)p(~FRAG)* | 13 | 12003.35 | 9.2133948 | 2.33E-03 | 10240.22 |
| *Phi(~FRAG * age2 + SEX)p(~FRAG)* | 17 | 12003.44 | 9.3050248 | 2.22E-03 | 10232.22 |
| *Phi(~FRAG + age2 * SEX)p(~FRAG)* | 15 | 12004.67 | 10.5388272 | 1.20E-03 | 10237.5 |
| *Phi(~age2 + SEX)p(~FRAG)* | 9 | 12008.49 | 14.3497765 | 1.78E-04 | 10253.42 |
| *Phi(~age2 * SEX)p(~FRAG)* | 11 | 12010.05 | 15.9197153 | 8.13E-05 | 10250.96 |

**Table S2.2.** Model averaged estimates of age (immature, adult), sex (female, male) and fragment specific survival.

| estimate | se | AGE | SEX | FRAG |
| --- | --- | --- | --- | --- |
| 0.821 | 0.020 | Ad | F | CH |
| 0.731 | 0.046 | Ad | F | FU |
| 0.747 | 0.037 | Ad | F | MB |
| 0.802 | 0.044 | Ad | F | ND |
| 0.833 | 0.020 | Ad | F | NG |
| 0.842 | 0.016 | Ad | M | CH |
| 0.759 | 0.044 | Ad | M | FU |
| 0.774 | 0.034 | Ad | M | MB |
| 0.825 | 0.040 | Ad | M | ND |
| 0.853 | 0.015 | Ad | M | NG |
| 0.391 | 0.102 | Im | F | CH |
| 0.288 | 0.104 | Im | F | FU |
| 0.490 | 0.235 | Im | F | MB |
| 0.582 | 0.291 | Im | F | ND |
| 0.459 | 0.104 | Im | F | NG |
| 0.437 | 0.109 | Im | M | CH |
| 0.328 | 0.115 | Im | M | FU |
| 0.531 | 0.225 | Im | M | MB |
| 0.621 | 0.269 | Im | M | ND |
| 0.506 | 0.104 | Im | M | NG |

**Table S2.3.** Survival rates averaged over fragments as used in this study.

|  | estimate |
| --- | --- |
| Male juvenile (σ_0m)_ | 0.485 |
| Female juvenile (σ_0f)_ | 0.442 |
| Male adult (σ_1m)_ | 0.811 |
| Female adult (σ_1f)_ | 0.787 |

*Appendix S3: Density-dependence in dispersal and settlement*

We included effects of population density on emigration probability (*d*) and the settlement probability (*P_s_*) of dispersing males such that emergent population equilibrium densities reflected currently observed densities. In RS, the parameter *b* that specifies the strength of demographic density-dependence is also used to model density-dependence in emigration and in settlement. The emigration reaction norm to density is modelled as:

$d=\frac{D_{0}}{1+e^{-\left( {bN}_{i,t}-\beta\right)\alpha}}$ eqn. S3.1

Here, *D_0_* is the maximum emigration probability, *β* is the inflection point of the function while *α* determines the slope at the inflection point. *N_i,t_* represents the population size in patch *i* at time *t*. We parameterized this function such that *d* ≈ 0 at low density and *d* ≈ 0.20 for females and *d* ≈ 0.15 for males at the density currently observed and *d* ≈ 0.70 and *d* ≈ 0.50 at high densities (i.e. approximating the maximum emigration probability *D*)

The settlement reaction norm to density is given by the function:

$P_{s}=\frac{1}{1+e^{-\left( {bN}_{i,t}-\beta_{s} \right)\alpha_{s}}}$ eqn. S3.2

Where *N_i,t_* is the number of individuals in patch *i* at time *t*, *β_s_* is the inflection point and *α_s_* determines the slope of the function at the inflection point. We estimated *β_s_* and *α_s_* such that *P_s_* ≈ 1 at low density and *P_s_* ≈ 0 at high density.

*Appendix S4: Sensitivity analysis*

The sensitivity analysis was performed on the following model parameters: fecundity (φ), juvenile survival (σ_0_), adult survival (σ_1_), and the maximum emigration probability (*D*). Parameter values were individually varied simultaneously for both sexes. Please note that in RangeShifter the emigration probability and the immigration probability are emergent properties of the model and therefore not eligible to be included in this analysis.

**Fig. S4.1** Results of the sensitivity analysis showing the change in mean final population size for each scenario relative to baseline predictions.

**Table S1.** Predictions obtained for each patch under the baseline scenario.

| Patch | Mean final abundance (number of birds ± SE) | Mean immigration rate (inds/year ± SE) | Mean probability of occupancy (± SE) |
| --- | --- | --- | --- |
| CH | 100.56 (± 0.969) | 1.49 (± 0.030) | 1.00 (± 0.000) |
| FU | 12.07 (± 0.422) | 1.71 (± 0.030) | 1.00 (± 0.000) |
| MA | 1.97 (± 0.165) | 0.49 (± 0.014) | 0.64 (± 0.018) |
| MS | 8.50 (± 0.292) | 1.83 (± 0.027) | 1.00 (± 0.000) |
| MW | 0.24 (± 0.062) | 0.06 (± 0.004) | 0.11 (± 0.008) |
| ND | 2.89 (± 0.203) | 0.83 (± 0.017) | 0.87 (± 0.005) |
| NG | 182.19 (± 1.270) | 1.64 (± 0.035) | 1.00 (± 0.000) |
| YA | 4.08 (± 0.253) | 0.85 (± 0.019) | 0.83 (± 0.018) |

*
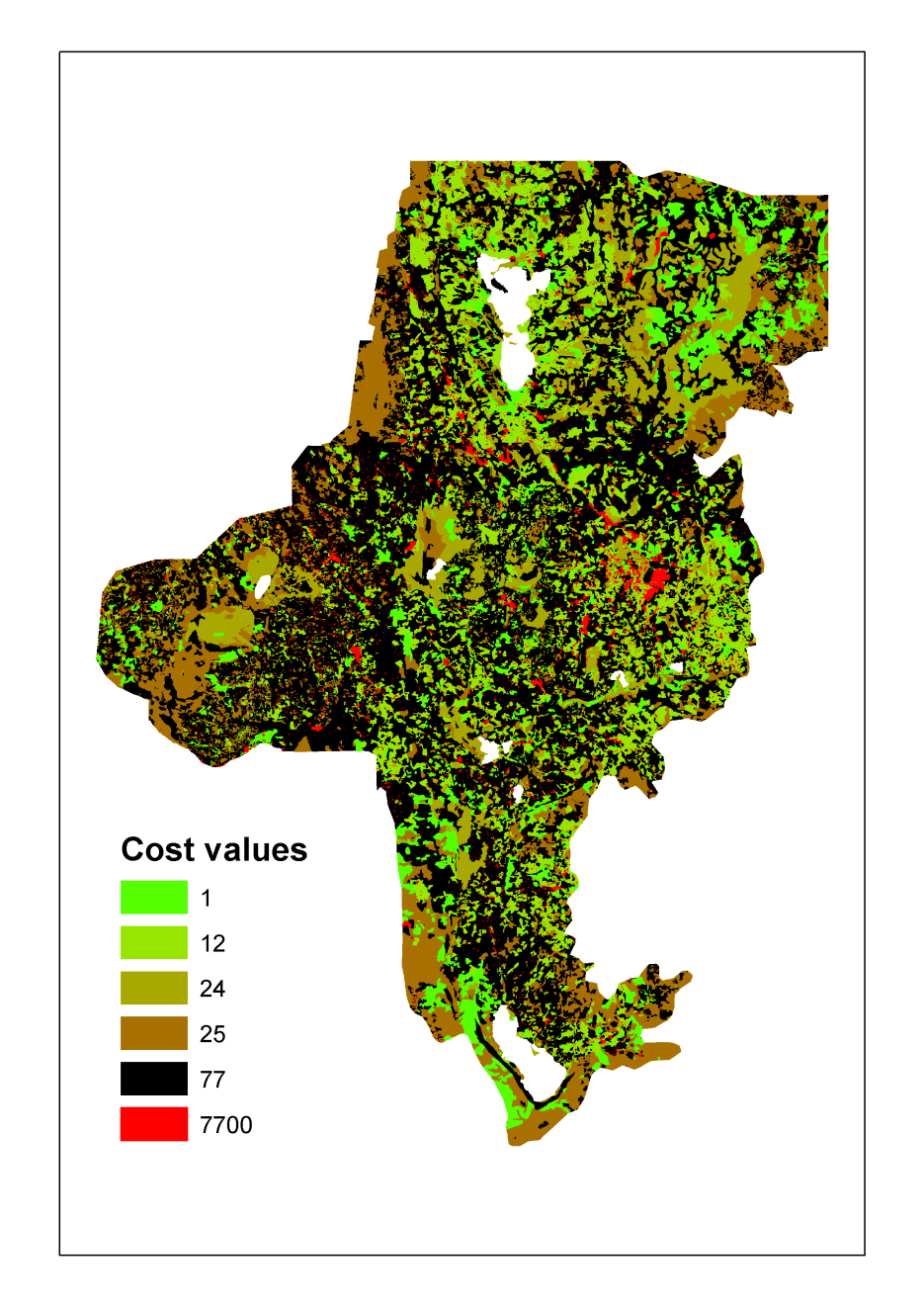
*

**Fig. S1.** Cost surface of the study area showing the original habitat patches in white.

**References**

Aerts, R., Thijs, K.W., Lehouck, V., Beentje, H., Bytebier, B., Matthysen, E., Gulinck, H., Lens, L., Muys, B., 2011. Woody plant communities of isolated Afromontane cloud forests in Taita Hills, Kenya. Plant Ecology 212, 639-649.

Burnham, K.P., Anderson, D.R., 2002. Model selection and multimodel inference: a practical information-theoretic approach. Springer Science & Business Media.

Callens, T., 2012. Genetic and demographic signatures of population fragmentation in a cooperatively breeding bird from south-east Kenya. Ghent University, Ghent.

Korfanta, N.M., Newmark, W.D., Kauffman, M.J., 2012. Long-term demographic consequences of habitat fragmentation to a tropical understory bird community. Ecology 93, 2548-2559.

White, G.C., Burnham, K.P., 1999. Program MARK: survival estimation from populations of marked animals. Bird study 46, S120-S139.
